# Supplementary figures and images for: Deep Eutectic Solvents as New Extraction Media for Flavonoids in Mung Bean
Source: Foods. 2024 Mar 1;13(5):777. doi: 10.3390/foods13050777 (PMC10931339; doi:10.3390/foods13050777)

## Supplementary Figure S1

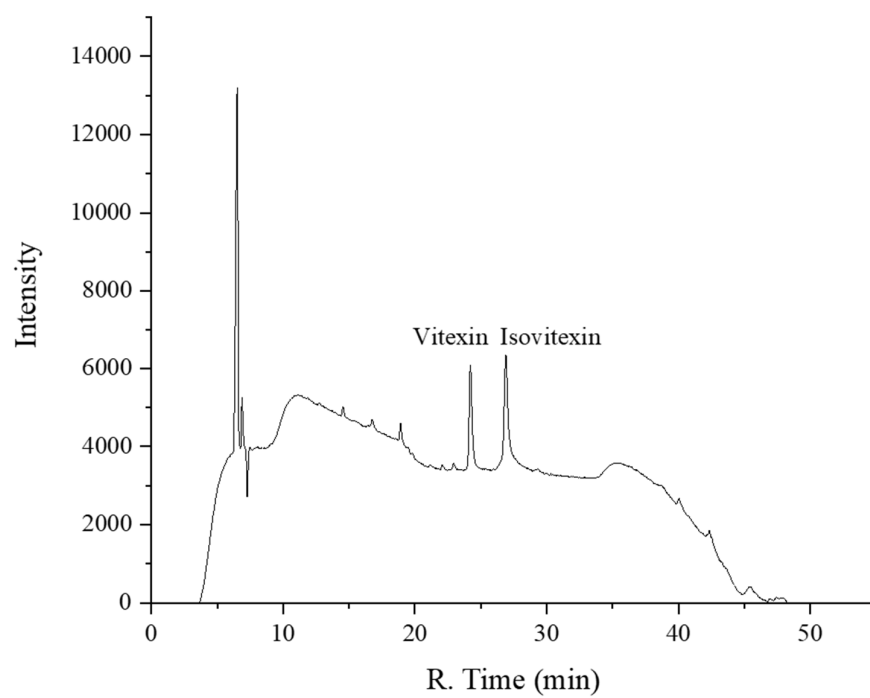

**Figure S1.** The chromatogram of mung bean flavonoids.

Supplement: Supplementary file 1 [file foods-13-00777-s001.zip › foods-2883501-supplementary.pdf]
